# Retraction: Evaluation of Tegaran Formula ZhenHua cytotoxicity against human cancer cell lines

**DOI:** 10.1371/journal.pone.0283065

**Published:** 2023-03-09

**Authors:** The PLOS ONE Editors

After this article [1] was published, a series of concerns were raised, including about the results, references, Competing Interests statement, and use of Tegaran as an anti-cancer therapy agent. *PLOS ONE* discussed the concerns with the authors, and a member of the journal’s Editorial Board then evaluated the article and comments provided by the authors. Although the authors’ comments clarified some of the reporting issues, the Section Editor advised that major concerns were not satisfactorily resolved in the author discussions. Specifically:

- The study design is not sufficient to address the study’s stated objective, “to evaluate the potential health effects of Tegaran Formula ZhenHua”.
- The methods and results were not reported in sufficient detail.
- QC analytical assessment of the product was not carried out in full.
- Cytotoxicity experimental results across a dose response curve and across timepoints were not reported, as would be needed to meet community standards for analyses of cytotoxic activity. The primary data for Tables 4, 5, and 6 were not provided.
- The clinical relevance of the study and its findings are unclear, due to concerns about the product’s bioavailability and the concentrations used in functional assays. Bioavailability assays were not conducted as part of this study, and the capsule shell did not fully dissolve at pH 2 or pH 6.8 and was manually removed.
- The total isoflavone content value presented in Table 3 was generated by summing the contents of genistein, glycitein, and daidzein only, and did not consider isoflavone glucosides present in the soy extract (the production and extraction processes were not reported in sufficient detail in article [1]).
- The data in Tables 4, 5, and 6 appear to suggest that the cytotoxic activities of Tegaran are generally greater than those of 13-MTD, which appears inconsistent with some sentences in the abstract and conclusions of article [1].
- In the discussion, reference 21 is cited in support of the following statement: “Although 13-MTD is considered to be the main ingredient of Tegaran according to the study that was performed by Zhenhua Yang”, however this reference does not appear to show that 13-MTD is a component of Tegaran.
- It is not clear how it was determined that 1 mg/ml of Tegaran corresponds to 20 μg/ml of active substance (13-MTD), as 13-MTD does not appear to be detected in the assays performed in article [1], 13-MTD does not appear to be specified in the list of ingredients in Table 1, and there do not appear to be references in article [1] that support the Tegaran composition.

In light of these unresolved issues, the *PLOS ONE* Editors concluded that the study design was inadequate to address the study aims, and the conclusions are not supported. Therefore, we retract this article.

PP, IV, and IP did not agree with the retraction. AM, IB, and VD either did not respond directly or could not be reached.

At the time of publication of this notice, the authors did not provide the underlying data and full results from the GC-MS analysis, or the individual-level data for S1-S10 Figs, in response to journal requests.

There are errors in the article’s Competing Interests statement. The correct Competing Interests statement is: The authors of this article are affiliated with Research Genetic Cancer Centre International GmbH and Research Genetic Cancer Centre International SA. There are no patents, products in development or marketed products to declare. This does not alter our adherence to PLOS ONE policies on sharing data and materials.
